# Outlines of Physiology; with an Appendix on Phrenology

**Published:** 1840-04

**Authors:** 


Art. V.—*Outlines of Physiology; with an Appendix on Phrenology.* By P. M. Roget, m.d., Secretary to the Royal Society, &c. First American Edition, revised, with numerous Notes.—*Philadelphia,* 1839. 8vo, pp. 516.

This volume contains the treatises on physiology and phrenology contributed by Dr. Roget to the new edition of the Encyclopædia Britannica. They have been republished separately in this country, and, from the influence of Dr. Roget's name we presume, they have been reprinted almost immediately in America. Numerous additions, several of them of much value, have been made, in the form of notes, by the American editor—we believe Professor Dunglison; and the treatise on phrenology, instead of preceding the other, as it did in the English edition, is now converted into an appendix to it. This we deem its right situation, as phrenology, after all, is but a branch of physiology, and, to be well studied, must be studied on exactly the same principles. The character of the work has been well given by the American editor: "The present volume contains a concise, well-written epitome of the present state of physiology, human and comparative; not, as a matter to be expected, the copious details and developments to be met with in larger treatises on the subject, but enough to serve as an accompaniment and guide to the physiological student." It is written in that clear and simple style which characterizes all Dr. Roget's productions; and it is seldom behind the present state of knowledge. There is one remarkable inaccuracy, however, to which our attention was directed by a correctional note. The ultimate structure of the brain and nerves is described according to the now antiquated observations of De la Torre, Monro, the Wenzells, Edwards, &c.; and no allusion whatever is made to the fact, now satisfactorily ascertained by the observations of Ehrenberg, Berres, Valentin, and others, of their tubular character. Moreover, we find the medulla oblongata spoken of as the special seat of sensation—a doctrine which few now uphold. The chapter on the vital powers, on the other hand, contains a most excellent and condensed view of the chief questions which this subject involves. We are not altogether satisfied that the general character of this treatise is suitable to the work for which it was written. We should have been disposed to have made such an essay either more popular, by omitting the less important details and more fully explaining others, or more scientific, by including such higher generalizations as may be considered sound and permanent. We should have thought, too, that the human physiology ought to have followed, instead of preceding the comparative. But we shall presume that Dr. Roget was not altogether left to his own choice in the matter, and that the editor of the Encyclopædia is the party responsible for the general character of the treatise.

